# Effect of Story Structure Instruction Based on Visual Analysis on Reading Comprehension Intervention for Dyslexic Students

**DOI:** 10.1155/2022/9479709

**Published:** 2022-08-29

**Authors:** Hanzhu Yang

**Affiliations:** University of Leeds, Leeds LS29JT, UK

## Abstract

The application of artificial intelligence (AI) technology in the field of dyslexia is becoming increasingly abundant. However, the content of related literature shows that there is still a lack of systematic and comprehensive research in this field at home and abroad. By outlining the development of AI technology, the meaning, causes, and classification of learning disabilities, the most representative studies on the application of AI technology in dyslexia education, including four aspects of diagnosis, intervention, assessment, and services are analyzed. The study finds that AI technology can improve the conditions suffering from dyslexia and dysgraphia, and can serve the education of dyslexic children as a technical tool to overcome dyslexia. By summarizing the effect of story structure teaching based on visual analysis on reading comprehension intervention for students with dyslexia, it can provide useful references and references for related research.

## 1. Introduction

Dyslexia is a specific type of learning disability caused by a neurological problem that manifests as difficulty with spelling, decoding, and word recognition. In 2012, the International Dyslexia Association reported that 15–20% of school-age children across the United States have dyslexia, and up to 20% of children have partial dyslexia. For some students, dyslexia may even persist into adulthood. Dyslexia has serious implications for student learning, causing a variety of impairments such as spelling disorders, difficulty decoding words, and lack of fluency in reading aloud, and may also lead to problems in organizing learning strategies, language development, and motor development [1–7].

The existing research [8–10] shows that if children with reading problems are diagnosed and intervened with at an early age, up to 70% of students can be freed from the problem through special education or remedial education programs. This shows the importance of appropriate early intervention for dyslexia [11–14]. This study proposes a study of the effects of story structure instruction based on visual analysis on reading comprehension intervention for students with dyslexia in order to provide a basis for promoting research and practice of dyslexia intervention methods in English language.

## 2. Definition of Relevant Concepts

### 2.1. Teaching Story Structure

Story structure is also known as story grammar, story composition, story mapping instruction, story mapping instruction, story schema, etc. This study defines story structure instruction as the presentation of important elements in a story by means of visual diagrams, including the main character, the situation, the main issue, what happens, and the ending of the story [15–18]. Readers improve their reading comprehension by mastering the elements in the story structure, establishing a story framework, and further analyzing the content of the text.

### 2.2. Dyslexia

According to the World Health Organization's definition, dyslexia (dyscalculia) is a state of reading or writing difficulty in which an individual is not different from other individuals in terms of general intellectual motivation, life circumstances, and educational conditions, and has no significant visual, hearing, or neurological impairments, but his or her reading or writing performance is significantly below what it should be at the appropriate age [19–23]. Dyslexia is the most predominant type of learning disability, accounting for more than 70% of all children diagnosed with a learning disability. Studies have shown that up to 10–30% of children in English-speaking countries have dyslexia. The number of children learning Chinese, who have dyslexia is around 3–5%.

### 2.3. Reading Comprehension

This study defines reading comprehension as the process by which a reader extracts information from written materials, and uses reading strategies to turn the text into the complex cognitive process of integrating clues from the chapter with prior knowledge and experience in order to understand the meaning of the text, including surface contextual understanding vs. deep contextual understanding. Surface contextual understanding is the understanding of the clear message of the story, and for students, the answers to the questions can be found directly in the original text [24–26]. Deep contextual understanding is the understanding of the implicit message in the story that students are required to integrate the content of the story or to respond with their own experiences, such as the cause-and-effect relationship of the story, the main idea of the article, and the theme of the story, etc. [25].

## 3. Study Design

### 3.1. Study Object

In this study, one male and one female, a total of two dyslexic students in the fifth grade of an elementary school, were selected as experimental study subjects. The reference criteria were as follows: first, normal intelligence; second, the phenomenon of falling behind in reading achievement; and third, parental consent for the students to participate in the experimental instruction.

The researcher learned the following about the subjects by asking the classroom teachers and English teachers of the two subjects.

Subject A, male, 13 years old, had normal intelligence. In the usual teaching of reading comprehension, the student read aloud not by words, but randomly according to his own ideas; reading speed was slow; he could not use the clues in the article to infer the content of the article, and thus could not understand the general idea of the article, and his reading comprehension always showed a low level.

Subject B, female, 13 years old, had normal hearing and vision as well as neurology. The student knew a high amount of words, but had high spelling errors and often added or subtracted letters from words; was able to read word by word or with finger assistance, but had inaccurate comprehension of the text and had great difficulty expressing herself in writing. In daily learning, the student has poor generalization skills and often generalizes; has significant difficulty integrating contextual information.

### 3.2. Experimental Hypothesis

In this study, story structure instruction was used as the independent variable in the experiment, and the reading comprehension development level of dyslexic students was used as the dependent variable. The research hypothesis was that story structure instruction could effectively promote the development of reading comprehension of dyslexic students.

### 3.3. Experimental Studies

The experiment was divided into three parts: a baseline period (A), an instructional intervention period (B), and a maintenance period (A′). The baseline period (A) lasted 2.5 weeks, consisted of two quizzes per week, for a total of five quizzes, and was designed to collect information about students' reading comprehension performance before the story structure instructional intervention. Students were asked to read the text themselves and were given a question-and-answer test without any prompting or feedback during the test period, in order to collect basic information about students′ reading comprehension before the intervention. The demonstration phase (B) lasted for 6 weeks, with two experiments per week, for a total of 12 experiments.  Table 1 shows the flow chart of the story structure instruction. In this phase, the researcher presented the “story train study sheet” after the students read the text aloud and guided them to add to the study sheet through a question and answer session. At the end of the instruction, students were given a reading comprehension quiz to collect changes in reading comprehension after the instructional intervention.

In the maintenance phase (A′), the test was administered two weeks after the end of the teaching period, for 2.5 weeks, twice a week. A total of 5 tests were administered to understand the effectiveness of the maintenance of the teaching experiment, no instruction was given during this period, and the same methods were used as in the baseline (A) phase.

### 3.4. Data Processing

#### 3.4.1. Visual Analysis

This study used the A-B-A′ experimental design in a single-subject experimental study, and visual analysis was the main method of data processing. The visual analysis mainly consisted of two parts, intrastage and interstage analysis. The following are the indicators used in this study.

*(1) Intrastage Analysis*

  Estimated convergence: the convergence line within the phase, i.e., the slope of the data path. Up (/) is noted as progressive (+); down (\) is noted as regressive (−); and horizontal (−) is noted as smooth (=).
  Convergence stability: the highest value of stage *x* the convergence stability criterion (20%) = acceptable stability range; the number of data points falling within the stability range of the convergence line ÷ total data points = convergence stability, above (including) 75%, which is stable; below 75%, which is unstable.
  Average: the average of all information points within the phase.
  Level range: the difference between the highest data point and the lowest data point within the phase.
  Level change: the difference between the last data point and the first data point within the phase.
  Level stability: the highest value of the stage × level stability standard (20%) = acceptable stability range; the number of data points falling within the stability range of the average level ÷ total data points = level stability, over (inclusive).

If it is 75%, it is stable; if it is less than 75%, it is unstable.

*(2) Interstage Analysis*

  Convergence trend and effect change: refers to the convergence line path and change between phases.
  Tendency to stable change: refers to the tendency to stable change between stages.
  Level change: the difference between the last data point of the previous period and the first data point of the latter period.
  Overlap percentage: the percentage of data points from the latter stage that falls within the range of data points from the former stage.

#### 3.4.2. C Statistics

The C-statistic can compensate for the lack of visual analysis by examining whether the data changes within and between stages reach a significant level. It is calculated by the formula:

(1) $$\begin{array}{c} C=1-\frac{\sum_{t=1}^{N-1}{\left(x_{t}-x_{t+1}\right)}^{2}}{2\sum_{i=1}^{N}{\left(x_{i}-x\right)}^{2}},\\ Sc=\sqrt{\frac{N-2}{\left(N-1\right)\left(N+1\right)}},\\ Z=\frac{C}{Sc}, \end{array}$$

In the formula, *x* represents the test score, $\bar{x}$ represents the mean, and *N* represents the number of experiments.

The degree of stability of the data points can be determined from the Z-values obtained from the C-statistics. If the *Z* value reaches a significant level, then it means that the data value of this stage has changed a lot; if the *Z* value does not reach the significant level, then it means that the data of this stage values vary a little.

## 4. Analysis of the Effect of Story Structure Teaching on the Overall Intervention of Reading Comprehension for Dyslexic Students

To explore the effect of story structure instruction on the overall intervention of reading comprehension for students with dyslexia, this study draws two a line graph of the change in overall reading comprehension scores for the six subjects was used to further ensure the reliability of the analysis by conducting visual analysis and C-statistical tests on the data. Reading comprehension included surface contextual understanding (out of 8) and deep contextual understanding (out of 8), with an overall reading comprehension score of 16 out of 16.

**Figure 1:**
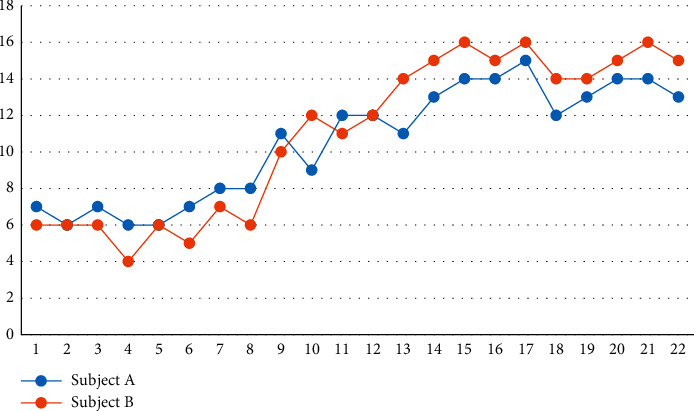
Line chart of reading comprehension for two subjects.

The overall reading comprehension scores of the two subjects in the three phases of baseline (A), instructional intervention (B), and maintenance (A′) are shown in Figure 1. The tests were 1–5 baseline period, 6–17 teaching intervention period, and 18–22 maintenance period.

As shown in Figure 1, the reading comprehension scores of both subjects showed an increasing trend, and story structure instruction was positively correlated with reading comprehension scores. In the baseline period, both subjects′ reading comprehension scores showed a low level, with little fluctuation in performance and stable reading comprehension performance. Subject A's performance was slightly higher than that of subject B, which might be related to subject A's better reading comprehension foundation. During the teaching intervention period, the reading comprehension scores of both subjects showed an increasing trend, with the highest score of 15 for subject A and the highest score of 16 for subject B, which was related to the seriousness of subject B's learning attitude. At the beginning of the instructional intervention, subject A's reading comprehension score was slightly higher than B's. As the instruction proceeded, subject B's reading comprehension score exceeded A's, and the two subjects developed their reading comprehension at slightly different rates. During the maintenance period, both subjects' first reading comprehension scores decreased slightly and then immediately rose to a higher level and showed a stable trend. The decrease in subject B's first reading comprehension score during the maintenance period was smaller than that of A, indicating that story structure instruction was more effective in maintaining subject B's reading comprehension.

Thus, it is clear from the line graph that both subjects′ reading comprehension scores improved as the instruction progressed, but there were interindividual differences in the speed and stability of development.

### 4.1. Analysis of the Effects of Story Structure Instruction on the Various Stages of Reading Comprehension Intervention for students with Reading Disabilities

Based on the scores of the two subjects′ overall reading comprehension scores in the three phases of baseline (A), instructional intervention (B), and maintenance (A′), the information analysis table for each phase of overall reading comprehension scores was tallied and plotted, as shown in Table 2.

According to Table 2, subject A took the reading comprehension test five times during the baseline period, with convergent stability of 100% and a steady downward trend in reading comprehension scores. The mean value was 6.40, and the performance remained at a low level. Level range 6-7 (+1), level change 7-6 (−1), and level stability was 100%, with scores remaining stable. There was no significant difference in data variation within the phase (*C* = −0.25, *Z* = −0.71, *p* > 0.05), indicating that in this phase subject A's reading comprehension scores did not change significantly and was ready for instructional intervention. After entering the instructional intervention phase, subject A completed a total of 12 reading comprehension tests, with convergent stability of 83% and a steady upward trend in reading comprehension scores. The mean value was 11.08, which was an increase in performance compared to the baseline period. Both the range and level change were 7–15 (+8), with level stability of 33% and a substantial increase in reading comprehension scores. The data change within the period reached a significant level of 0.01 (*C* = 0.76, *Z* = 2.87, *p* < 0.01), indicating that the reading comprehension scores of the A students in this period were significantly improved and the teaching effect was significant.

Subject A took a total of five reading comprehension tests during the maintenance period with convergent stability of 100% and a steady upward trend in reading comprehension scores. The mean value was 13.20, and the scores remained at a high level. The level range was 12–14 (+2), the level change was 12-13 (+1), and the level stability was 100%, with small changes in reading comprehension scores. There was no significant difference in data change within the period (*C* = 0.46, *Z* = 1.31, *p* > 0.05), indicating that the reading comprehension scores of subject A were stable and well maintained during this period. Subject B took a total of five reading comprehension tests during the baseline period and showed a decreasing trend in reading comprehension scores. The tendency stability was 60%, showing an unstable state, which may be related to the lower maximum value in this stage. Convergent stability is obtained by multiplying the highest value of the stage by the stability criterion to obtain an acceptable stability range, and the proportion of data points falling within the range is calculated to determine whether the convergence is stable or not. Lower convergent stability was derived due to the narrow range of acceptable stability resulting from the lower maximum value in the baseline period of subject B. However, the data did not show an improving trend and could enter the teaching intervention period. The mean value for this period was 5.60, keeping the scores low. The level range was 4–6 (+2), the level change was 6-6 (0), the level stability was 80%, and the scores remained stable. There was no significant difference in the data change within the phase (*C* = −0.25, *Z* = −0.71, *p* > 0.05), indicating that there was no significant change in the performance of subject B in this phase to intervene in teaching.

After entering the instructional intervention phase, subject B completed a total of 12 reading comprehension tests with convergent stability of 83% and a steady upward trend in reading comprehension scores. The mean value was 11.58, which was an increase in performance compared to the baseline period. Both the range and level change of the standards were 5–16 (+11), with level stability of 33% and a substantial increase in reading comprehension scores. The data change within the period reached a significant level of 0.01 (*C* = 0.90, *Z* = 3.39, *p* < 0.01), indicating that the reading comprehension scores of subject B improved significantly during this period and the teaching effect was significant. Subject B took a total of 5 reading comprehension tests during the maintenance period, with convergent stability of 100% and a stable upward trend in reading comprehension scores. The mean value was 14.80, and the performance remained at a high level. Level range 14–16 (+2), level change 14–15 (+1), level stability of 100%, and small changes in reading comprehension scores. There was no significant difference in the data change within the stage (*C* = 0.46, *Z* = 1.31, *p* > 0.05), indicating that the reading comprehension scores of subject B were stable and had a good maintenance effect in this stage.

### 4.2. Analysis of the Effect of Story Structure Teaching on the Interstage Intervention of Reading Comprehension for Dyslexic Students

Based on the scores of the two subjects' overall reading comprehension scores in the baseline (A), instructional intervention (B), and maintenance (A′) periods, the interstage information analysis table for overall reading comprehension scores was tallied and plotted, as shown in Table 3.

As can be seen from Table 3, from the baseline period to the instructional intervention period, subject A's reading comprehension scores first decreased and then increased, and the tendency stability all showed stability. The change in level was 6-7 (+1), indicating that the reading comprehension scores of subject A showed an immediate increase after the instructional intervention. The overlap percentage was 8%, and subject A scored at the baseline period level in only 1 out of 12 tests during the intervention period, indicating that the story structure instruction had a favorable effect on subject A's reading comprehension performance. The data change between phases was significantly different (*C* = 0.87, *Z* = 3.80, *p* < 0.01), indicating a significant improvement in subject A's reading comprehension performance between phases. From the instructional intervention period to the maintenance period, subject A's reading comprehension scores showed a steady upward trend. The change in level was 15-12 (−3), indicating a significant decrease in subject A's reading comprehension scores after withdrawal from instruction. The overlap percentage was 100%, indicating that all five reading comprehension scores of subject A in the maintenance period were within the range of changes in the intervention period and that the story structure instruction had a good maintenance effect. The data change between phases was significantly different (*C* = 0.74, *Z* = 3.26, *p* < 0.01), indicating a significant difference in subject A's reading comprehension scores between phases.

From the baseline period to the instructional intervention period, subject B's reading comprehension scores first decreased and then increased, and tended to change in stability from unstable to stable, with a level change of 6-5 (−1), indicating that after the intervention instruction, subject B's reading comprehension scores did not show an immediate increase. The overlap percentage was 16%, and subject B scored at the baseline period level on only 2 out of 12 tests during the intervention period, indicating that story structure instruction had a favorable effect on subject B's reading comprehension scores. The data change between phases was significantly different (*C* = 0.93, *Z* = 4.06, *p* < 0.01), indicating a significant improvement in subject B's reading comprehension performance between phases. From the instructional intervention period to the maintenance period, subject B's reading comprehension scores showed a steady upward trend. The change in level was 16-14 (−2), indicating a decrease in subject B's reading comprehension scores after withdrawal from instruction. The overlap percentage was 100%, indicating that all five reading comprehension scores of subject B in the maintenance period were within the range of changes in the intervention period and that the story structure instruction had a good maintenance effect. The data change between phases was significantly different (*C* = 0.90, *Z* = 3.94, *p* < 0.01), indicating a significant difference in subject B's reading comprehension scores between phases.

### 4.3. Comparison of Learning Effects of Story Structured Instructional Materials and Reading Comprehension Test Scores

**Figure 2:**
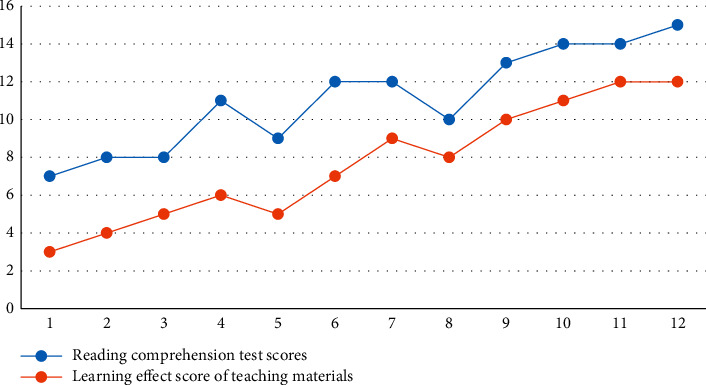
Subject A story structure line diagram of the change of learning effect and reading comprehension test results.

**Figure 3:**
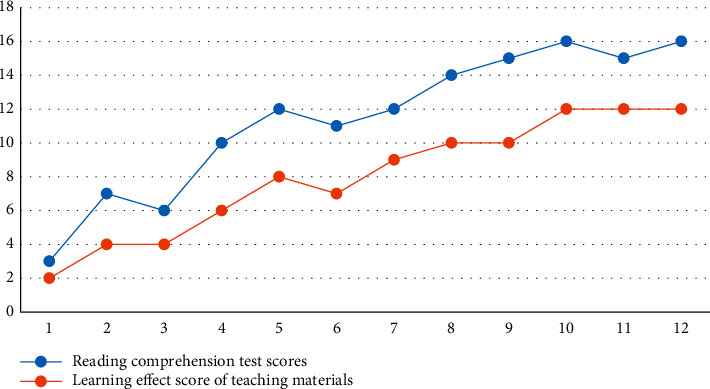
Subject B story structure line diagram of the change of learning effect and reading comprehension test results.

To further illustrate the trends of the learning effects of story-structured instructional materials and reading comprehension test scores, the line graphs of the learning effects of instructional materials scores and reading comprehension test scores were plotted separately for two subjects during the instructional intervention period, as shown in Figures 2 and 3.

According to Figure 2, during the instructional intervention period, subject A's learning effectiveness scores for instructional materials and reading comprehension test scores both showed an increasing trend, and the changes in both were basically the same. The reading comprehension test scores increased when the learning effectiveness scores of instructional materials increased and decreased when the learning effectiveness scores of instructional materials decreased. For example, in the second, fourth, sixth, ninth, and tenth experiments, subject A's learning effectiveness scores increased, and so did her reading comprehension test scores. In the 5th and 8th experiments, subject A's learning effectiveness scores decreased and his reading comprehension test scores also decreased.

According to Figure 3, during the instructional intervention period, subject B's learning effectiveness scores for instructional materials and reading comprehension test scores both showed an increasing trend, and the changes in both were basically the same. The reading comprehension test scores increased when the learning effectiveness scores of instructional materials increased and decreased when the learning effectiveness scores of instructional materials decreased. For example, in the second, fourth, fifth, seventh, eighth, and tenth experiments, subject B's learning effectiveness scores increased, and so did his reading comprehension test scores. In the 6th experiment, subject B's learning effectiveness scores decreased and his reading comprehension test scores also decreased.

## 5. Conclusion

Combining the results of the line graph analysis of the two subjects, the learning effectiveness of the instructional materials of the two subjects during the instructional intervention period. The results scores showed high consistency with the reading comprehension test scores, both showing an increasing trend. Instructional material study learning effect scores increased, reading comprehension test scores increased accordingly, and learning effect scores for instructional materials decreased. Reading comprehension test scores also decreased. Thus, it can be shown that two subjects, who received story structure instruction after a change in the learning effect of story structure instruction, caused a change in reading comprehension test scores. The learning effect of story-structured instructional materials was consistent with reading comprehension test scores.

## Figures and Tables

**Table 1:** Teaching process of story structure.

Teaching program	Teaching procedure	Teaching time (min)
Lead the engine	Show the story title, predict the story content, and stimulate the students' interest in learning	5

Read the text	Focus on letting students understand the content of the story, reduce the reading burden of students with dyslexia, and lead students to read words and explain the meaning of new words. Students read the text aloud under the guidance of the teacher	5

Story structure teaching	Show the story train learning list, explain or review the five structural elements of the story. Use questions and answers to encourage students to find out the corresponding structure of the article, and fill in the answers in the story train learning list	15

Extended activities	Students should retell the story content or deduce the story content according to the content already filled in	5

Reading comprehension test	Show the reading comprehension test, the students read the article by themselves, and then collect the answers, and do not give them any hints or feedback	10

**Table 2:** Reading comprehension of the overall performance analysis table of each stage.

Stage order	Subject A			Subject B		
	Baseline	Intervention	Maintenance	Baseline	Intervention	Maintenance
	A1	B1	A1′	A2	B2	A2′
Stage length	5	12	5	5	12	5
Estimated trend	—	—	—	—	—	—
	(−)	(+)	(+)	(−)	(+)	(+)
Trend stability	5/5	10/12	5/5	3/5	10/12	5/5
	100%	83%	100%	60%	83%	100%
	Stabilize	Stabilize	Stabilize	Instability	Stabilize	Stabilize
Average value	6.40	11.08	13.20	5.60	11.58	14.80
Level range	6-7	7–15	12–14	4–6	5–16	14–16
	(+1)	(+8)	(+2)	(+2)	(+11)	(+2)
Level change	7-6	7–15	12–13	6-6	5–16	14-15
	(−1)	(+8)	(+1)	(0)	(+11)	(+1)
Level stability	5/5	4/12	5/5	4/5	4/12	5/5
	100%	33%	100%	80%	33%	100%
C	Stabilize	Instability	Stabilize	Stabilize	Instability	Stabilize
Z	−0.71	2.87^*∗∗*^	1.31	−0.71	3.39^*∗∗*^	1.31

*Note.* ^*∗*^*p* < 0.05, *Z* test significance level 0.05;  ^*∗∗*^*p* < 0.01 and *Z* test significance level 0.01.

**Table 3:** Reading comprehension overall achievement interstage analysis table.

Stage comparison	Subject A		Subject B	
	A1/B1	B1/A1′	A2/B2	B2/A2′
Towards the trend and the effect of the change	—	—	—	—
	−+	++	−+	++
Trend to stability change	Stable to stable	Stable to stable	Instable to instable	Stable to stable
Level change	6–7	15–12	6–5	16–14
	(+1)	(−3)	(−1)	(−2)
Percentage of overlap	1/12	5/5	2/12	5/5
	8%	100%	16%	100%
C	0.87	0.74	0.93	0.90
*Z*	3.80^*∗∗*^	3.26^*∗∗*^	4.06^*∗∗*^	3.94^*∗∗*^

*Note.* ^*∗*^*p* < 0.05, *Z* test significance level 0.05; ^*∗∗*^*p* < 0.01 and *Z* test significance level 0.01.

## Data Availability

Data are available on request from the corresponding author.
